# A natural knockout of the *MYO7A* gene leads to pre‐weaning mortality in pigs

**DOI:** 10.1111/age.13068

**Published:** 2021-05-06

**Authors:** M. F. L. Derks, H.‐J. Megens, W. L. Giacomini, M. A. M. Groenen, M. S. Lopes

**Affiliations:** ^1^ Animal Breeding and Genomics Wageningen University & Research Wageningen The Netherlands; ^2^ Topigs Norsvin Research Center Beuningen The Netherlands; ^3^ Topigs Norsvin Curitiba Brazil

**Keywords:** animal breeding, animal welfare, loss‐of‐function, mortality, *MYO7A*, usher syndrome

## Abstract

The pig breeding system provides a unique framework to study recessive defects and the consequence on the phenotype. We examined a commercial synthetic Duroc population for recessive defects and identified a haplotype on chromosome 9 significantly affecting pre‐weaning mortality. To identify the causal variant underlying the mortality, we examined sequence data of four carrier animals and 21 non‐carrier animals from the same population. The results yield a strong candidate causal stop‐gained variant (NM_001099928.1:c.541C>T) affecting the *MYO7A* gene in complete linkage disequilibrium with the lethal haplotype. The variant leads to an impaired (p.Gln181*) MYO7A protein that truncates 2032 amino acids from the protein. We examined a litter from a carrier sow inseminated by a carrier boar. From the resulting piglets, two confirmed homozygous piglets suffered from severe balance difficulties and the inability to walk properly. The variant segregates at a carrier frequency of 8.2% in the evaluated population and will be gradually purged from the population, improving animal welfare. Finally, this 'natural knockout' will increase our understanding of the functioning of the *MYO7A* gene and provides a potential model for Usher syndrome in humans.

In pig breeding, recessive defects often go unnoticed as the frequency of the associated variant is often low and the effect on the population marginal (Harlizius *et al*. 2020). Nevertheless with the growing availability of genotype and sequence data, even relatively low frequency defects can be identified and the causal variant can be mapped (Derks *et al*. 2019).

In this study, we examined 14 160 (10 402 boars, 3758 sows) genotyped animals (Porcine 50K SNP chip) from a synthetic Duroc population. The position of the SNPs was based on the *Sus* *scrofa* 11.1 reference genome (Warr *et al*. 2020). We retained 43 026 SNPs with a call rate >0.90, a minor allele frequency >0.01, and without strong deviation from Hardy–Weinberg equilibrium (*P* > 1 × 10^−6^) using plink v1.90b6.5 software (Purcell *et al*. 2007). Next, we phased and imputed the genotypes using beagle 5.0 software using default settings except that the effective population size parameter was set to 150 (Browning *et al*. 2018). Subsequently, we applied a sliding window approach to identify haplotypes with a deficit in homozygosity similarly as applied in Derks *et al*. (2019). Specifically, we moved along the genome in steps of 500 kb, 1 Mb, and 1.5 Mb to test for haplotypes showing a deficit in homozygosity applying an exact binomial test (*P* < 0.05).

One strong candidate region was discovered at the start of chromosome 9 (6–15 Mb, Table S1). One haplotype (11.0–12.0 Mb, frequency: 4.1%) showed the most significant deficit in homozygosity as 24 homozygotes were expected but only one was observed (Table 1). To provide further evidence of a recessive lethal variant segregating on this haplotype, we examined 31 past carrier‐by‐carrier litters and observed a significant 23% decline in pre‐weaning survival (Table 2). The 31 litters produced a total of 316 piglets (281 born alive, Table S2). From the 281 piglets born alive, 81 did not survive the lactation period (29%, Table S3). Sixty‐six out of 81 died within the first 5 days, while some lived until the end of the lactation period. However, it is not clear whether the animals that lived until the end of the lactation period (23 days) were homozygous for the haplotype. In addition, from 38 piglets (that died during the lactation period), a cause of death was submitted into the system. For 24 out of 38 piglets that died early from the carrier‐by‐carrier crosses ‘nervous disease’ was marked as the cause of death (Table S4). Nervous disease is generally indicated when the piglets have difficulty maintaining balance, and show ‘shaky’ behaviour.

**Table 1:** SSC9 haplotype characteristics.

Position, Mb	SSC9: 11.0–12.0
Number of markers	34
No. haplotype carriers	1156
Homozygotes expected (HWE)	23.6
Homozygotes observed	1
Exact binomial test	5.83e‐11
Carrier frequency %	8.2%
C × C matings	31
Genes in region	*ACER3, B3GNT6, CAPN5, MYO7A, PAK1*

Expected and observed homozygotes for the haplotype on SSC9, also genes in the window are shown.

**Table 2:** Carrier‐by‐carrier litters show 23% decrease in pre‐weaning survival compared to carrier‐by non‐carrier litters.

Status	No. litters	Avg. totalborn	Avg. liveborn	Farrowing survival %	Weaning survival %
N × N	4556	10.29	9.36	90.61	89.55
C × N	777	10.31	9.28	89.28	90.41
C × C	31	10.19	9.06	87.90	**69.99** *

The weaning period corresponds to 21 days.

* *P* < 0.01.

Significant results are indicated in bold.

Next, we examined sequence data of four carrier and 21 non‐carrier animals from the breed under study to identify the putative causal variant. We applied similar analysis as described in Derks *et al*. (2019). In short, we removed adapter and low quality sequences using Trimmomatic 0.39 (Bolger *et al*. 2014), mapped the reads using bwa‐mem 0.7.15 (Li & Durbin 2009), and used samtools 1.9 to sort, merge, and index the bam files (Li *et al*. 2009). We performed variant calling using freebayes v1.3.1 with the following settings ‐‐min‐base‐quality 10, ‐‐min‐alternate‐fraction 0.2, ‐‐haplotype‐length 0, ‐‐min‐alternate‐count 2 (Garrison & Marth 2012). We discarded variants with PHRED quality <20. The resulting sequence variants were functionally annotated using the Ensembl Variant Effect Predictor pipeline (build 99) (McLaren *et al*. 2016). In addition, we called structural variants using the smoove v0.2.2 pipeline within the haplotype region (±2 Mb; Pedersen 2020). The sequence statistics for the 25 samples under study are presented in Table S5.

To identify putative causal variants, we performed an LD analysis between the sequence variants and the haplotype using Plink with the following settings ‐‐chr‐set 18, ‐‐r2, ‐‐ld‐window‐r2 0.8 (Purcell *et al*. 2007). The analysis yielded 172 candidate causal variants. Four variants affected the coding sequence of genes, three were synonymous (predicted to have low impact) and one was predicted to have high impact (Table S6). The high impact variant is a stop‐gained variant (g.11280403C>T) in the *MYO7A* gene. All four carrier animals were heterozygous for the stop‐gained variant, while the non‐carriers from the same breed were homozygous for the reference allele. The variant (NM_001099928.1:c.541C>T) results in a stop codon at position p.Gln181* in the protein (Fig. 1a,b), resulting in an impaired and truncated MYO7A protein (Fig. 1c). The mutant MYO7A protein lacks the final 2032 amino acids and will be likely to impair the function of the protein completely. We also examined the presence of structural variations, but no candidate causal structural variants were discovered in the haplotype region.

**Figure 1:**
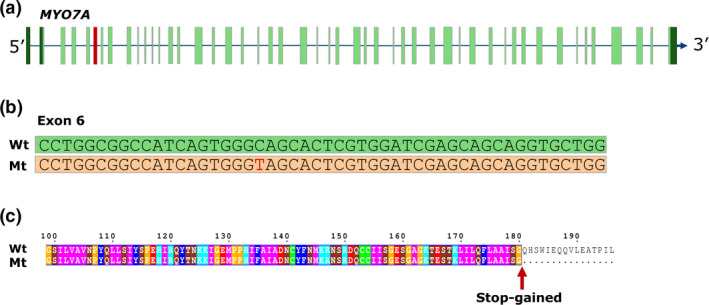
(a) *MYO7A* gene model. The location of the affected 6th exon is indicated in red, the UTRs are indicated in dark green. (b) Illustration of the stop‐gained variant. Figure shows wildtype and mutant exon (c) Alignment of the mutant (Mt) and wildtype (Wt) MYO7A protein sequence. The variant induces a premature stop codon leading to a truncated MYO7A protein.

To further assess the consequence of the haplotype (and the candidate *MYO7A* stop‐gained variant), we examined a carrier‐by‐carrier litter that farrowed on 1 September 2020 in Parana State, Brazil. The litter resulted in 11 born alive piglets and one still born. Two piglets died 2 days later on 3 September and both piglets showed nervous system issues, i.e. shaking behaviour, difficulties maintaining balance, and difficulty walking (Video S1). The whole litter was genotyped on the 25K SNP chip and imputed to the 50K Porcine Beadchip. Both ‘shaky’ piglets were homozygous for the haplotype on chromosome 9. From the remaining nine littermates, five were heterozygous for the haplotype and four were non‐carriers (including the stillborn piglet; Table S7).

MYO7A is a member of the myosin superfamily of proteins (Li *et al*. 2020). Myosins are actin‐based motor molecules exhibiting ATPase activity and are thereby involved in intracellular movements. Myosins exhibit divergent tails that bind to membrane compartments, which are then moved relative to actin filaments (Li *et al*. 2020).

Variants in *MYO7A* are associated with Usher syndrome (deaf‐blindness) and other types of deafness in humans (OMIM: 276903; Koenekoop *et al*. 1993). Variants in the *MYO7A* gene cause Usher syndrome type 1B. Affected individuals are usually deaf at birth and undergo progressive retinal degeneration during life. In addition, patients usually exhibit constant vestibular dysfunction leading to balance difficulties and they experience problems walking (Mathur & Yang 2015). Note that Usher syndrome is not lethal in humans. However, in pigs, the affected piglets will be ‘selected out’ or euthanized (possibly mainly due to balance difficulties), which explains the absence of homozygous animals in a ‘mature’ population. In mice, *MYO7A* knockouts show head shaking, deafness, retinal defects, and reduced male fertility (Williams 2008). Moreover, a missense mutation was described in the Doberman pinscher dog breed associated with bilateral deafness and vestibular dysfunction (Webb *et al*. 2019). In the retina, *MYO7A* plays an important role in the renewal of the outer photoreceptor disks, essential for the distribution and migration of retinal pigment epithelial melanosomes and phagosomes, and in the regulation of opsin transport in retinal photoreceptors (Mathur & Yang 2015). In the inner ear, *MYO7A* is important for the differentiation, morphogenesis, and organization of cochlear hair cell bundles (Mathur & Yang 2015). In addition, *MYO7A* is involved in hair‐cell vesicle trafficking of aminoglycosides ototoxicity (Richardson *et al*. 1997). Together, this evidence indicates at least one functional copy of *MYO7A* is required for normal hearing. However, shaker‐1 knockout mice did not show retinal degradation, but do show hyperactivity, head‐tossing, and circling due to vestibular dysfunction (Gibson *et al*. 1995). Hence, knockouts of this gene leads to a wide range of syndromes mainly affecting hearing, vision, and vestibular dysfunctions.

In human and mouse, dominant loss‐of‐function variants in *MYO7A* have been described that cause deafness (Liu *et al*. 1997). The syndrome leads to a form of progressive neurosensory hearing loss with post‐lingual onset and some affected individuals exhibit mild vestibular symptoms. We tested whether the carrier sow (from the C × C litter above) reacted to noise and observed that the sow did not react to sound at all, but she was definitely not blind (she responded to visual stimuli). The onset of deafness could not be determined and needs further investigation. Nevertheless, hearing loss could be an interesting phenotype in pigs and is of high interest to the pig breeding industry because it can also be associated with abnormal behaviour in domestic animals (Strain 2015).

Usher syndrome in humans is mainly caused by missense variants and inframe deletions affecting various essential amino acids in the MYO7A protein (Rong *et al*. 2014). In addition, the dominant and recessive deafness forms are also caused by mostly missense variants, but affecting different amino acids compared to the Usher syndrome type (Liu *et al*. 1997). Together, this shows a complex interplay between the genomic variants and the resulting phenotype. Variants that cause an extremely truncated MYO7A protein (as observed in this study) are rare in humans, and therefore, this variant in pigs might be of high interest.

In conclusion, we report a natural knockout of the *MYO7A* gene, providing an interesting model to study Usher syndrome. We show that piglets homozygous for a stop‐gained variant in the *MYO7A* gene suffer from balance difficulties which usually results in the death within the first 10 days after birth during the weaning period. Furthermore, we have indications that the variant might lead to deafness in (older) heterozygous carrier animals, but this needs further investigation. Our study shows again that the growing resources of genomic resources obtained in the breeding industry provides an excellent dataset to study deleterious variants and their consequence on the phenotype.

## Conflict of interest

M.F.L.D., W.L.G., and M.S.L. are employees of Topigs Norsvin Research Center, a research institute closely related to one of the funders (Topigs Norsvin). All authors declare that the results are presented in full and as such present no conflict of interest. The other Breed4Food partners Cobb Europe, CRV, Hendrix Genetics, declare to have no competing interests for this study.

## Funding

This research was funded by the STW‐Breed4Food Partnership, project number 14283: From sequence to phenotype: detecting deleterious variation by prediction of functionality This study was financially supported by NWO‐TTW and the Breed4Food partners Cobb Europe, CRV, Hendrix Genetics and Topigs Norsvin. The use of the HPC cluster was made possible by CATAgroFood (Shared Research Facilities Wageningen UR).

## Disclaimer

The data used in this study have been obtained as part of routine data collection from Topigs Norsvin breeding programmes, and not specifically for the purpose of this project. Therefore, approval of an ethics committee was not mandatory. Sample collection and data recording were conducted strictly according to the Dutch law on animal protection and welfare (Gezondheids‐ en welzijnswet voor dieren).

## Supporting information

**Table S1**. Haplotypes showing deficit in homozygosity.
**Table S2**. Overview of 31 C × C litters.
**Table S3**. Survival of 281 piglets born alive from 31 C × C matings.
**Table S4**. Cause of death description for piglets from C × C litters.
**Table S5**. Mapping and coverage statistics from WGS samples from the synthetic Duroc population under study (including the carrier status).
**Table S6**. Genomic variation in high LD with the SSC9 haplotype.
**Table S7**. *MYO7A* haplotype of the C × C litter including two affected individuals (farrowing date: September 1st 2020).

**Video S1**. Video showing both affected individuals after birth.

## Data Availability

50K Genotypes and WGS variants (VCF) are available at the Open Science Framework repository: https://osf.io/9zhm6/.
