# Supplementary material for: A natural knockout of the MYO7A gene leads to pre‐weaning mortality in pigs
Source: Anim Genet. 2021 May 6;52(4):514–7. doi: 10.1111/age.13068 (PMC8360181; doi:10.1111/age.13068)
Supplement: Supplementary file 3 [file AGE-52-514-s001.docx]

**Video S1:** Video showing both affected individuals after birth. The video clearly indicates balance difficulties observed for the two homozygous animals.
